# Editorial: Microglia as a Therapeutic Target for Brain Repair: Opportunities and Challenges

**DOI:** 10.3389/fncel.2022.877567

**Published:** 2022-03-16

**Authors:** Kai Zhou, Robert Adam Harris, Xianli Shen

**Affiliations:** ^1^Henan Neurodevelopment Engineering Research Center for Children, Children's Hospital Affiliated to Zhengzhou University, Zhengzhou, China; ^2^Applied Immunology and Immunotherapy, Department of Clinical Neuroscience, Center for Molecular Medicine, Karolinska Institutet, Karolinska University Hospital, Stockholm, Sweden; ^3^Department of Cancer Immunology and Virology, Dana-Farber Cancer Institute, Boston, MA, United States; ^4^Department of Immunology, Harvard Medical School, Boston, MA, United States

**Keywords:** microglia, brain repair, neuroinflammation, microglial depletion, CNS diseases

Microglia play essential roles in physiological functions of the central nervous system (CNS) and in pathological progression of CNS diseases (Kettenmann et al., 2011; Streit et al., 2014). Microglial dysfunctions directly contribute to some CNS diseases that are currently difficult to cure, such as colony-stimulating factor-1 receptor (CSF-1R)-related leukoencephalopathy caused by a single gene mutation in microglia (Konno et al., 2018). Moreover, microglia highly express a variety of Alzheimer's disease (AD) risk genes that are critical for the pathogenesis of AD (Hansen et al., 2018). The modulation of microglial functional phenotypes is therefore emerging as an area of interest for potential therapeutic strategy in order to treat various CNS diseases, including neurodevelopmental disorders, neurodegenerative diseases and brain tumors. This Research Topic focuses on the opportunities and challenges of treating various CNS diseases by targeting microglia. We address three specific topics: (1) Reversing dysfunctional microglial functions in CNS diseases; (2) Alleviating neuroinflammation in CNS diseases; (3) Seeking new ways to target microglia effectively. Dedicated to this promising treatment strategy for CNS diseases, our Research Topic includes three original research articles, two reviews, and one perspective article. Here, we briefly introduce these publications in this editorial.

Hermann and Gunzer provided their perspective on two advanced toolsets accelerating our understanding of the roles of microglia playing in both physiological and pathological settings. The first toolset comprises *brain imaging and analysis technologies*, including two-photon microscopy, confocal microscopy and software solutions for imaging analysis. These technologies increase our understanding of the dynamic morphological changes of microglia, reflecting different microglia activities. The second toolset is the *microglial depletion strategies*, including pharmacologically and genetic modification approaches. Microglial depletion has been a useful tool to increase understanding of both the physiological and pathological roles of microglia in the CNS (Han et al., 2017), and can be developed as a therapeutic strategy to treat various diseases.

For a long time, microglia were recognized for their phagocytosis of apoptotic cells in the brain (Ferrer et al., 1990). Ischemic stroke, the major type of stroke, occurs due to a lack of blood flow in part of the brain, followed by shortage of oxygen and nutrients which subsequently leads to cell death within minutes in the ischemic core. Huang et al. investigated whether activating Big Potassium (BK) channels in microglia could improve neurological outcomes after ischemic stroke. First, they detected a decreased BK channel expression in the ischemic stroke mouse model. Next, they demonstrated that activation of the BK channel could promote microglial phagocytosis. Furthermore, they showed that activation of BK channels could reduce neuronal apoptosis and improve motor functions after ischemic stroke. Improved microglial phagocytosis following activation of BK channels is thus beneficial in the acute phase of ischemic stroke.

Microglia acquire distinct phenotypes in response to diverse microenvironmental cues (Keane et al., 2021). Li et al. reviewed the signaling pathways regulating microglial phenotypic transitions. They first discussed the widely used traditional M1/M2 classification and its limitations. Next, they summarized the modulators and signaling pathways regulating microglial phenotypic transitions that have therapeutic potential for various CNS diseases. AD, the most common form of dementia, is characterized by dysregulated microglial activation, inflammatory changes, impaired Aβ plaque clearance and neuronal loss which ultimately leads to cognitive decline (Heneka et al., 2015). Zhang et al. comprehensively reviewed the pathophysiological roles of microglia in AD. Firstly, they discussed the phagocytic capacity of microglia of Aβ under different conditions and the possible signaling pathways regulating Aβ clearance. Secondly, they discussed how over-activated microglia contribute to chronic inflammation and promote AD progression. Next, they discussed the associations between dysfunctional microglia and tau pathology, and between dysfunctional microglial mitophagy and microglial phagocytosis. Finally, they discussed microglial modification therapeutics for AD based on targeting of these signaling pathways.

Apart from AD, neuroinflammation with microglia as the central player is a critical process in other CNS diseases. Targeting the pro-inflammatory responses mediated by microglia has thus become a promising therapeutic strategy. A study by Liu et al. revealed the underlying mechanisms of propofol in inhibiting pro-inflammatory microglial activation. They demonstrated that propofol could effectively suppress microglial activation by modulating miR106 via down-regulating PI3K/Akt signaling activity. Ham et al. investigated the anxiolytic effect of N-Allyl-2-[(6-butyl-1,3-dimethyl-2,4-dioxo-1,2,3,4-tetrahydropyrido[2,3-d]pyrimidin-5-yl)sulfanyl]acetamide (G721-0282). They first demonstrated that G721-0282 could relieve anxious behaviors in mice following induction of chronic unpredictable mild stress. Furthermore, they demonstrated that the anxiolytic effect of G721-0282 is attributed to the inhibition of IGFBP3-mediated neuroinflammation through inhibiting CHI3L1.

Together, the articles included in this Research Topic describe recent advances and findings in targeting microglia and modulating neuroinflammation in various CNS diseases, which provide valuable insights into the research field of neuroscience and promote the development of potential therapeutic strategies for these devastating CNS diseases.

## Author Contributions

KZ, RH, and XS wrote the manuscript, acted as editors to this Research Topic, and selected the articles described herein. All authors contributed to the article and approved the submitted version.

## Conflict of Interest

The authors declare that the research was conducted in the absence of any commercial or financial relationships that could be construed as a potential conflict of interest.

## Publisher's Note

All claims expressed in this article are solely those of the authors and do not necessarily represent those of their affiliated organizations, or those of the publisher, the editors and the reviewers. Any product that may be evaluated in this article, or claim that may be made by its manufacturer, is not guaranteed or endorsed by the publisher.

## References

[B1] Ferrer I. Bernet E. Soriano E. del Rio T. Fonseca M. (1990). Naturally occurring cell death in the cerebral cortex of the rat and removal of dead cells by transitory phagocytes. Neuroscience 39, 451–458. 10.1016/0306-4522(90)90281-8 2087266

[B2] Han J. Harris R. A. Zhang X. M. (2017). An updated assessment of microglia depletion: current concepts and future directions. Mol. Brain 10, 25. 10.1186/s13041-017-0307-x 28629387 PMC5477141

[B3] Hansen D. V. Hanson J. E. Sheng M. (2018). Microglia in Alzheimer's disease. J. Cell Biol. 217, 459–472. 10.1083/jcb.201709069 29196460 PMC5800817

[B4] Heneka M. T. Carson M. J. El Khoury J. Landreth G. E. Brosseron F. Feinstein D. L.. (2015). Neuroinflammation in Alzheimer's disease. Lancet Neurol. 14, 388–405. 10.1016/S1474-4422(15)70016-5 25792098 PMC5909703

[B5] Keane L. Cheray M. Blomgren K. Joseph B. (2021). Multifaceted microglia - key players in primary brain tumour heterogeneity. Nat. Rev. Neurol. 17, 243–259. 10.1038/s41582-021-00463-2 33692572

[B6] Kettenmann H. Hanisch U. K. Noda M. Verkhratsky A. (2011). Physiology of microglia. Physiol. Rev. 91, 461–553. 10.1152/physrev.00011.2010 21527731

[B7] Konno T. Kasanuki K. Ikeuchi T. Dickson D. W. Wszolek Z. K. (2018). CSF1R-related leukoencephalopathy: a major player in primary microgliopathies. Neurology 91, 1092–1104. 10.1212/WNL.0000000000006642 30429277 PMC6329328

[B8] Streit W. J. Xue Q. S. Tischer J. Bechmann I. (2014). Microglial pathology. Acta Neuropathol. Commun. 2, 142. 10.1186/s40478-014-0142-6 25257319 PMC4180960

